# Aspirin in the Treatment of Cancer: Reductions in Metastatic Spread and in Mortality: A Systematic Review and Meta-Analyses of Published Studies

**DOI:** 10.1371/journal.pone.0152402

**Published:** 2016-04-20

**Authors:** Peter C. Elwood, Gareth Morgan, Janet E. Pickering, Julieta Galante, Alison L. Weightman, Delyth Morris, Mark Kelson, Sunil Dolwani

**Affiliations:** 1 Cochrane Institute of Primary Care and Public Health, Cardiff University, Cardiff, United Kingdom; 2 Hywel Dda University Health Board, Llanelli SA14 8QF, United Kingdom; 3 Department of Psychiatry, University of Cambridge, Cambridge, United Kingdom; 4 Specialist Unit for Review Evidence, Cardiff University, Cardiff, United Kingdom; University of Oxford, UNITED KINGDOM

## Abstract

**Background:**

Low-dose aspirin has been shown to reduce the incidence of cancer, but its role in the treatment of cancer is uncertain.

**Objectives:**

We conducted a systematic search of the scientific literature on aspirin taken by patients following a diagnosis of cancer, together with appropriate meta-analyses.

**Methods:**

Searches were completed in Medline and Embase in December 2015 using a pre-defined search strategy. References and abstracts of all the selected papers were scanned and expert colleagues were contacted for additional studies. Two reviewers applied pre-determined eligibility criteria (cross-sectional, cohort and controlled studies, and aspirin taken after a diagnosis of cancer), assessed study quality and extracted data on cancer cause-specific deaths, overall mortality and incidence of metastases. Random effects meta-analyses and planned sub-group analyses were completed separately for observational and experimental studies. Heterogeneity and publication bias were assessed in sensitivity analyses and appropriate omissions made. Papers were examined for any reference to bleeding and authors of the papers were contacted and questioned.

**Results:**

Five reports of randomised trials were identified, together with forty two observational studies: sixteen on colorectal cancer, ten on breast and ten on prostate cancer mortality. Pooling of eleven observational reports of the effect of aspirin on cause-specific mortality from colon cancer, after the omission of one report identified on the basis of sensitivity analyses, gave a hazard ratio (HR) of 0.76 (95% CI 0.66, 0.88) with reduced heterogeneity (P = 0.04). The cause specific mortality in five reports of patients with breast cancer showed significant heterogeneity (P<0.0005) but the omission of one outlying study reduced heterogeneity (P = 0.19) and led to an HR = 0.87 (95% CI 0.69, 1.09). Heterogeneity between nine studies of prostate cancer was significant, but again, the omission of one study led to acceptable homogeneity (P = 0.26) and an overall HR = 0.89 (95% CI 0.79–0.99). Six single studies of other cancers suggested reductions in cause specific mortality by aspirin, and in five the effect is statistically significant. There were no significant differences between the pooled HRs for the three main cancers and after the omission of three reports already identified in sensitivity analyses heterogeneity was removed and revealed an overall HR of 0.83 (95% CI 0.76–0.90). A mutation of PIK3CA was present in about 20% of patients, and appeared to explain most of the reduction in colon cancer mortality by aspirin. Data were not adequate to examine the importance of this or any other marker in the effect of aspirin in the other cancers. On bleeding attributable to aspirin two reports stated that there had been no side effect or bleeding attributable to aspirin. Authors on the other reports were written to and 21 replied stating that no data on bleeding were available.

**Conclusions and Implications:**

The study highlights the need for randomised trials of aspirin treatment in a variety of cancers. While these are awaited there is an urgent need for evidence from observational studies of aspirin and the less common cancers, and for more evidence of the relevance of possible bio-markers of the aspirin effect on a wide variety of cancers. In the meantime it is urged that patients in whom a cancer is diagnosed should be given details of this research, together with its limitations, to enable each to make an informed decision as to whether or not to take low-dose aspirin.

**Systematic Review Protocol Number:**

CRD42015014145

## Introduction

Despite significant advances in diagnosis and treatment in recent decades, cancer is still one of the main causes of morbidity and mortality worldwide. It claims more than 81,000 males and 74,000 females every year in the UK alone, the crude annual mortality rate being about 250 deaths in every 100,000 people [1]. Much effort is now being focused on ‘targeted cancer therapies’, that is, drugs that interfere with specific molecules involved in cancer cell growth and cell survival, but as yet there have been few successes.

There is convincing evidence that regular low-dose aspirin not only reduces vascular disease incidence and mortality [2,3], but also reduces the incidence and mortality of colorectal and other cancers [4–7]. Furthermore, there is growing evidence which suggests that aspirin, used as an adjuvant treatment following a diagnosis of cancer, may reduce metastatic spread and may increase the survival of patients with cancer.

Chan et al (2009) [8], Langley (2011) [9,10] and others have pointed out that effects of aspirin on certain biological mechanisms justify an expectation of benefit from aspirin used as an adjunct treatment of patients with cancer. These effects include an interruption of tumour growth, a retardation of metastatic spread, an inhibition of angiogenesis, enhancements of both DNA mismatch repair and cellular apoptosis and an abrogation of invasiveness. Benefit from treatment with aspirin is therefore not unexpected.

Our aim in what follows is to provide a comprehensive systematic review and meta-analysis of the available evidence on the effects of aspirin used as an adjunct treatment of cancer in the reduction of mortality and metastatic spread.

## Methods

The review protocol was registered in PROSPERO (registration number CRD42015014145). In reporting we have followed the PRISMA guidelines [11].

In December 2015 observational and interventional studies in Medline and Embase were searched using a pre-defined strategy with indexed descriptors and keywords including “aspirin”, “acetylsalicylic acid”, “cancer” “tumour”, “neoplasm”, “mortality”, “death”, “adverse effect”, “bleed”. The search was limited to human studies in peer-reviewed journals and conference abstracts. Reference lists of the included studies were also searched and recent conference proceedings scanned and topic experts contacted for additional studies.

Studies were selected for inclusion in meta-analyses if (a) the studied population comprised patients diagnosed with cancer; (b) aspirin was taken regularly after cancer diagnosis independently of whether it had been taken before diagnosis; (c) they were case-control studies, cohort studies or controlled trials; and (d) cause-specific mortality was available. All-cause cancer mortality, incidence of metastases and adverse effects were noted but were not criteria for selection.

Two reviewers independently excluded reports that did not meet inclusion criteria based on title and abstract. Full published reports were obtained for the remainder, and inclusion criteria were applied.

The origin of the patient group and other details in each report were examined, and if there appeared to be two reports based on the same patients, if the evidence required for the meta-analyses was not clear, or if important items were missing, the author(s) was contacted and asked for clarification. Authors of all the papers were also asked whether they had data on gastrointestinal or other bleeding, and if this had been a concern at any time. All these processes were conducted by one of the authors and were checked as appropriate by another author.

The methodological quality of the included studies was assessed and graded independently by two authors using the Newcastle-Ottawa Scale [12]. Differences in grading of reports on a nine point scale, were discussed and agreed.

Meta-analyses were conducted grouping the studies according to study design: intervention and observational studies. Subgroup analyses were conducted according to cancer types, key mutations and whether or not patients had taken aspirin only after diagnosis.

The summary statistics derived in the meta-analyses were either a hazard ratio or a risk ratio each with 95% confidence intervals. The analyses were carried out using the statistical package STATA. The inverse-variance method was used to weight the individual studies and provide the pooled estimate of effects. A 'random effects' model was used throughout to incorporate an estimate of between-study variation into the calculation of common effects. Funnel plots were created to highlight outlying studies and look at publication bias. Publication bias was assessed using Egger's test [13]. Sensitivity analyses were performed to assess the influence of individual studies on the combined hazard ratios. Heterogeneity was assessed using the Q statistic and investigated by repeating the meta-analyses excluding, first low scoring studies and then, if substantial heterogeneity was still present, outlying studies, identified by the sensitivity analyses were omitted.

## Results

**Fig 1:**
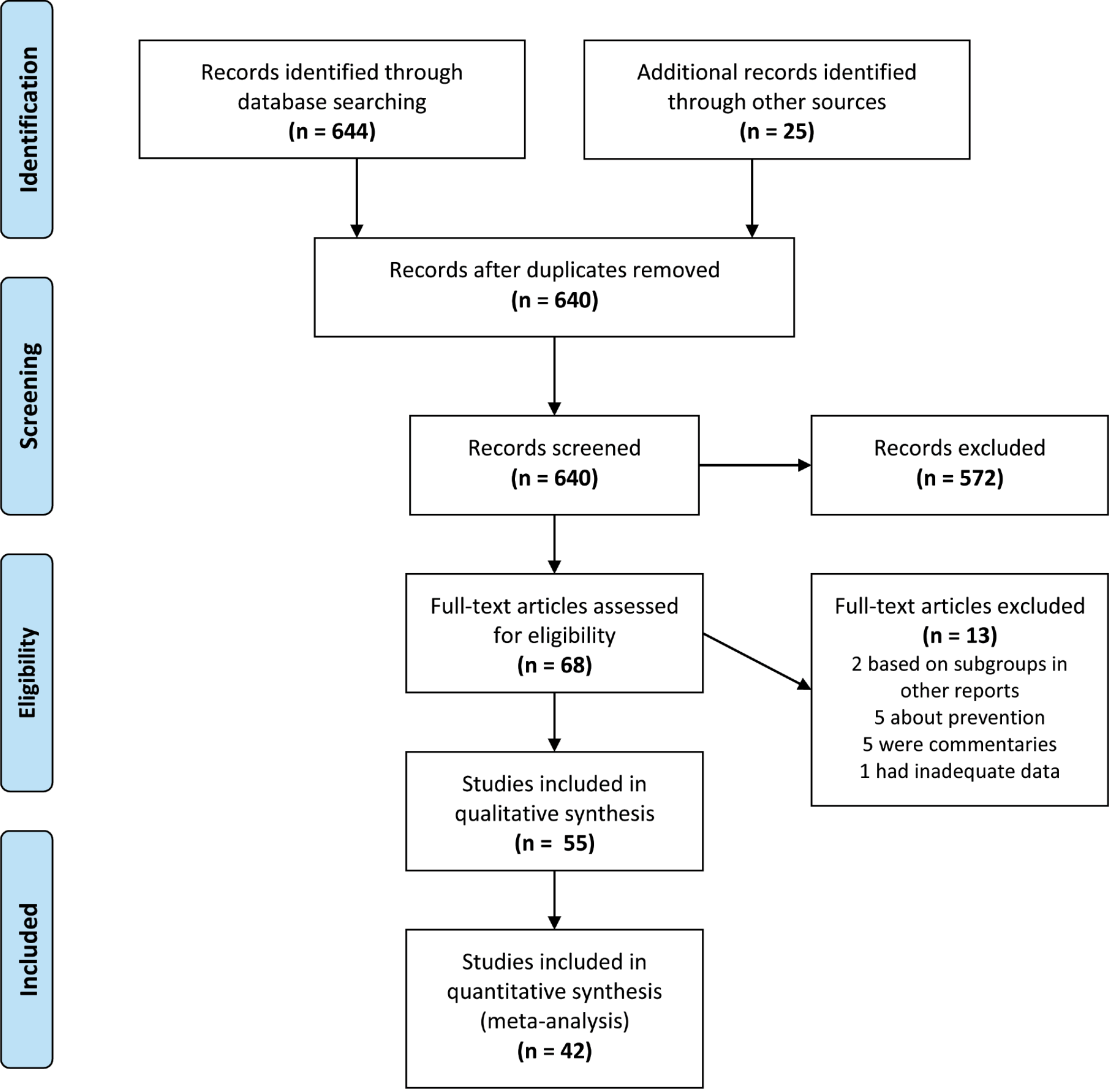
Prisma flow diagram.

The literature search identified 373 reports and following omissions of duplicates and irrelevant reports, 42 were found to be relevant and gave sufficient data to be included in meta-analyses. We present a summary diagram in Fig 1 showing the selection process.

Table 1 summarises a few basic features of the papers included in the meta-analyses together with those upon which further investigations are based. A final column gives an assessment of quality of the studies, judged according to the Newcastle-Ottawa Scale [12].

**Table 1:** Details of the studies.

Authors	Source	Design	Number of aspirin users and non-users. Duration of follow-up	Deaths in aspirin users and all-cause deaths	Comment	Grade
**Randomised controlled trials**						
Rothwell et al. [14]	Five early vascular trials	Randomised for vascular reduction	17,285 subjects randomised	385 deaths on aspirin, 402 deaths on placebo		RCT
Lipton et al. [15]	Series of patients	Ad hoc randomisation	57 patients randomised, F-U 24 months	Life table analysis, Numbers N.A.		RCT
LeBeau et al. [16]	Series of patients	Ad hoc randomisation	303 patients randomised, F-U 18 months	152 deaths on aspirin, 147 deaths on placebo		RCT
Cregan et al. [17]	Series of patients	Ad-hoc randomisation of patients with renal cancer	176 patients randomised, F-U 8.8 months	52 total deaths on aspirin, 56 deaths on placebo		RCT
Liu et al. [18]	Sequence of patients	Randomised by admission to ward	445 users, 1153 non-users, F-U 5 years	217 deaths in users, 685 deaths in non-users		RCT
**Reports of colorectal cancer**						
Bastiaannet et al. [19]	Eindhoven Cancer Registry	Cohort of 4481 patients with cancer	3305 users, 1176 non-users, F-U N.A.	114 CRC deaths in users, 610 deaths in non-users	Most appear to have had aspirin, not other NSAIDs	**9**
Bains et al [20]	Cancer Registry of Norway	Cohort of 25644 patients with cancer	6109 users, Non users N.A.	1172 CRC deaths in users, 6356 CRC deaths in non-users, 2088 total deaths in users, 7595 total deaths in non-users	Conference presentation	**7**
Cardwell et al. [21]	UK Clinical Practice Research Datalink	Nested case-control in a cohort of 4794 patients with cancer	Numbers N.A. Mean F-U 7.2 years	395 CRC deaths in users, 1164 deaths in non-users, 700 total deaths in users, 1514 total deaths in non-users		**9**
Chan et al. [8]	US Nurses and Health Professionals Cohorts	Cohort of patients with cancer	549 users, 730 non-users. Median F-U 11.8 years	81 CRC deaths in users, 141 CRC deaths in non-users, 193 total deaths in users, 287 total deaths in non-users	Varied dose of aspirin judged by frequency	**8**
Coghill et al. [22]	Seattle Cancer Family Register	Cohort of patients with cancer	234 users, 293 non-users. Mean F-U 8 years	37 events in users, 72 events in non-users		**9**
Din et al [23]	Series of cases of cancer	Case-control selected patients from a trial cohort	354 users, 526 non-users. F-U 1 years	125 deaths in users, 761 in non-users	NSAIDS, but data for aspirin given	**6**
Domingo et al. [24]	Series of patients	Cohort study	125 users, 771 non-users. F-U N.A.	22 deaths in users, 174 in non-users	Incidence and all-cause mortality in relation to PIK3CA state	**8**
Fuchs et al. [25]	Series of patients	Cohort study	72 users, 830 users. Mean F-U 2.4 years	Numbers of deaths N.A.		**5**
Goh et al. [26]	Series of patients	Cohort study	92 users, 634 non-users. F-U ‘long term’	21 CRC deaths in users, 160 CRC deaths in non-users		**9**
Liao et al. [27]	Nurses HS and Health Professionals Cohorts	Selected cohort of patients	337 users, 627 non-users. Mean F-U 5 years	68 CRC deaths in users, 122 CRC deaths in non-users		**7**
McCowan et al. [28]	Database of residents	Cohort of selected new patients	1340 users, 1650 non-users, F-U 11 years	420 CRC deaths in users, 601 CRC deaths in non-users, 897 total deaths in users, 1101 total deaths in non-users		**9**
Ng et al. [29]	Series of patients	Cohort study	75 users, 725 non-users. F-U 5 years	19 CRC recurrence in users, 21, CRC recurrence in non-users., 14 total deaths in users, 146 total deaths in non-users		**7**
Reimers et al [30]		Cohort of study of cancer patients	178 users, 784 non-users. F-U N.A.	68 deaths in users, 380 deaths in non-users	HLA class 1 antigen groups amalgamated	**9**
Sun et al. [31]	US Nurses and Health Professionals cohorts	cohort of selected cancer patients	931 subjects. Other details N.A. F-U 28 years	931 incident cases. Detailed numbers N.A.		**3**
Walker et al. [32]	UK GP Research Database	Cohort of selected patients	476 users, 10141 non-users. Median F-U 1.7 years	192 total deaths in users, 3910 total deaths in non-users		**9**
Zanders et al [33]	Eindhoven Cancer Registry	Cohort of selected patients with diabetes	490 users, 156 non-users. F-U 1.5 years	Numbers N.A.	Diabetic patients	**9**
**Reports of breast cancer**						
Barron et al [34]	Ireland National Cancer Registry	Cohort of 12507 patients with cancer	764users, 4540 non-users. F-U 7.4 years	50 breast cancer deaths in users, 311 breast cancer deaths in non-users, 311 total deaths in users, 459 total deaths in non-users		**7**
Blair et al [35]	Iowa Women’s Health Study	Cohort of 591 women with cancer	472 users, 120 non-users, F-U 15 years	26 breast cancer deaths in users, 22 breast cancer deaths in non-users, 57 total deaths in users, 44 total deaths in non-users		**8**
Bowers et al [36]	A Centre for Cancer Care	Cohort of 440 women with cancer	159 users, 281 non-users. F-U N.A.	Number of deaths not available	NSAIDs. 81% were aspirin	**7**
Cronon-Fenton [37]	Population based cohort in Denmark	Cohort study of 34188 patients	Median F-U 7.1 years	Numbers N.A.	Conference report	**6**
Frazer et al. [38]	Database of residents	Cohort of 4627 women	1244 users, 3383 non-users. F-U 16 years	252 breast cancer deaths in users, 563 breast cancer deaths in non-users, 577 total cancer deaths in users, 1225 total cancer in non-users		**8**
Holmes et al. [39]	US Nurses Health Study	Cohort of 4164 women	Number of users N.A. 5521 non-users. F-U n.a.	109 breast cancer deaths in users, 56 breast cancer deaths in non-users		**8**
Holmes et al. [40]	National Cancer Registry	Nested case-control within 27426 women	1661 users, 3322 non-users. F-U up to 5 years	395 breast cancer deaths in users, 750 breast cancer deaths in non-users		**9**
Kwan et al. [41]	Cohort of cancer patients	Cohort of 2292 women	Total 2292 women. Mean F-U 2.5 years	41 recurrent cancers in users, 209 recurrent cancers in non-users	NSAIDs	**8**
Murray et al. [42]	UK Clinical Practice Research Datalink	Nested case-control study	1173 users, 1173 non-users. Mean F-U 6.9 years	262/1435 cancer deaths in users, 1056/5697 cancer deaths in non-users		**9**
Wernli et al. [43]	Cohort of cancer survivors	Cohort of 3058 selected patients with breast cancer	541 users of NSAIDs, 2517 non-users of NSAIDs. F-U 6 years approx.	7 breast cancer deaths in users, 141 breast cancer deaths in non-users, 37 total deaths in users, 383 total deaths in non-users	NSAIDs	**7**
**Reports of prostate cancer**						
Assayaq et al [44]	UK National Cancer Data Repository	Cohort of 11779 newly diagnosed patients	Numbers in users N.A. F-U 5.4 years	801 cancer deaths in users, 992 cancer deaths in non-users, 1816 total deaths in users, 1686 deaths in non-users		**9**
Caon et al. [45]	Patient series	Cohort of newly 3851 diagnosed patients	509 users, 2428 non-users, F-U 7 years	194 cancer deaths in users, 904 cancer deaths in non-users		**8**
Choe et al. [46]	Patient registry	Cohort of 5955 patients	1817 users, 1736 no relevant drugs. Medium F-U 70 months	36 cancer deaths in users, 298 cancer ca deaths in non-users		**7**
Daugherty et al. [47]	Screened cohort	Cohort of patients with cancer	Numbers N.A. Medium F-U 5 years	136 cancer deaths		**8**
Dhillon et al. [48]	US Health Professionals cohort	Cohort study	1579 users, 1926 non-users, F-U up to 18 years	177 cancer deaths. Details N.A.		**8**
Flahavan [49]	Irish National Cancer Registry	Cohort study of 2936	1131 users, 1805 non-users. Median F-U 5.5 years	Numbers of deaths N.A.		**8**
Grytli et al. [50]	Cancer Registry of Norway	Cohort of selected patients	1279 users, 3515 non-users. Mean F-U 39 months	504 cancer deaths in users		**9**
Jacobs, Chun et al. [51]	Series of patients	Cohort study	45 users, 29 non-users. Mean F-U 56.6 months	6 cancer deaths in users, 8 cancer deaths in non-users		**6**
Jacobs, Newton et al. [52]	Prospective cohort of subjects	New cancer patients. Also ‘High-risk’ patients	3600 users, 3058 non-users, F-U up to 9 years	134 cancer deaths in users, 112 cancer deaths in non-users		**8**
Stock et al. [53]	Cancer Registry	cohort of selected cancer patients	419 users, 1200 non-users. Maximum F-U 120 months	115 cancer deaths in users, 338 cancer deaths in non-users	NSAIDs	**9**
**Reports of other cancers**						
Nagle et al [54]	Series of women with ovarian cancer	Cohort study of 1305 women with ovarian cancer	Numbers N.A., F-U 4.9 years	834 deaths		**6**
Fontaine et al. [55]	Series of patients with lung cancer	Cohort study of women with lung cancer	412 users, 1353 non-users, F-U 7.5 years	Numbers of deaths N.A.		**7**
Pastore et al. [56]	Series of patients with bladder cancer	Cohort of 574 patients with bladder cancer	98 users, 56 non users F-U 2 years	Numbers of deaths N.A.		**8**
Chae et al. [57]	536 patients with mixed cancers	Cohort of 536 women with mixed cancers	54 users, 482 non-users. Median F-U 8.8 months	Numbers of deaths N.A.		**4**
Chae et al [58]	Patients with relapsed/refractory chronic lymphocytic leukaemia	Retrospective study of 280 patients with chronic lymphocytic leukaemia	37 users, 17 non-users. Median F-U 4 years	Numbers of deaths N.A.		**5**
MacFarlane et al [59]	Series of 2392 patients with head and neck and oesophageal cancers	Cohort study of 2392 patients with oesophagus cancer; 1195 with head & neck cancer	1197 oesophagus, F-U 9 months, 1195 head & neck, F-U 35 months	965 oesophagus cancer deaths, 509 head & neck deaths cancer. Details N.A.		**7**
**Reports included in other tables**						
Algra & Rothwell [4]	Based on a literature search	Overviews of 6 RCTs; 150 case-control studies, and 45 cohort studies	In case-control studies. Followed for up to 20 years	245 in RCTs; 141,577 in case-control studies, 41,575 in cohort studies	Details on metastatic spread in RCTs and in 5 observational studies	**9**
Ljung et al. [60]	National Cancer Registry	Selected patient cohort	3424users, 23104 non-users, F-U 5 years	Numbers with lymph node metastases. Numbers N.A.		**8**
Kothari et al. [61]	Two cancer centres	Series of selected 999 patients with colon cancer	49 users, 136 non-users. Mean F-U 54 months	Detail of deaths N.A.		**7**

CI: confidence interval; CRC: Colorectal cancer;; F-U: Follow-up; N.A.: not available; NSAID: non-steroidal anti-inflammatory drug; RCT: randomised controlled trial; RR: risk ratio.

In the tables that follow we summarise the individual papers and report the results of meta-analyses and when available we give data for both cause-specific mortality and all-cause mortality.

### Aspirin, specific and overall mortality

Our search identified four reports of randomised trials, together with a report of pooled trials [14] (Table 2). The *ad hoc* trials [15–18] were small and the results did not achieve significance. The report by Rothwell et al (2012) [14] describes a 6.5 year follow-up of five early vascular trials, during which time 987 new cancers developed. In these, aspirin was associated with a reduction in cancer deaths (HR 0.71; 95% confidence limits (CI) 0.57, 0.90). The effect of aspirin in all five trials together is homogeneous (P = 0.30), but this result should be taken with caution as clinical heterogeneity such as differences between the design of the studies, the patient populations etc. may be too great to justify the pooling of results.

**Table 2:** Mortality in randomized trial patients with cancer who took aspirin versus placebo/no-aspirin.

Study	Design	Cancer	Aspirin/none	Outcome	Numbers of outcome events aspirin/placebo	Effect of aspirin (95% CI)
Rothwell Wilson [14]	Pooled analysis of five RCTs	All solid cancers	385.402	Cancer deaths	385,402	HR 0.71 (0.57,0.90)
				All deaths	N.A.	HR 0.81 (0.65, 1.00)
Lipton [15]	RCT	Colorectal	35,22	Cause-specific mortality	N.A.	HR 0.65 (0.02–18.06) ^a^
Lebeau [16]	RCT	Lung	153/150	Cause-specific mortality	152,147	HR 1.01 (0.81–1.27) ^a^
Cregan [17]	RCT	Renal	89/87	Cause-specific mortality	56,57	HR 0.91 (0.63–1.31) ^a^
Liu et al [18]	RCT ^b^	Oesophagus	445/658	Cause-specific mortality	217,388	HR 0.83 (0.68, 1.01)
Cause specific mortality: **HR 0.85** (0.74–0.97) *heterogeneity p = 0*.*30*						
All-cause mortality: **HR 0.81** (0.65–1.00) *Rothwell et al [14] alone*						

CI: confidence interval; HR Hazard Ratio; HR: hazard ratio; RCT: randomised controlled trial.

^a^Hazard ratios taken from Langley [20]

^b^Randomisation was achieved by admitting patients to two different wards in which aspirin and placebo were given.

In Table 3 data from observational studies are listed within three main groups: 16 on colorectal cancer [8,19–32], 10 on breast [34–43] and ten on prostate cancers [44–53], and then data relating to six other cancers [54–59]. A column contains comments of possible relevance on some of the reports of possible relevance.

**Table 3:** Results of aspirin treatment of cancer in observational studies.

Study	Aspirin/none	Mortality	Deaths (aspirin, no aspirin)	Results (95% CI)	Comment
**Colorectal cancer**					
Bastiaannet et al [19]	275/ 1176	All-cause	114, 610	HR 0.77 (0.63, 0.95)	Frequent use HR 0.70 (0.57, 0.88)
Bains et al [20]	6109/19535	Specific	1172, 6356	HR 0.53 (0.50, 0.57)	
		All-cause	2088, 7595	HR 0.71 (0.68, 0.75)	
Cardwell et al [21]	1005/ 2365	Specific	395, 1164	HR 0.99 (0.86, 1.15)	
Chan et al [8]	549/1279	Specific mort	81, 141	HR 0.71 (0.53, 0.95)	Specific: Only post diagnosis: RR 0.53 (0.33, 0.86). Pre and post: RR 0.89 (0.59, 1.35)
		All-cause	193, 287	HR 0.79 (0.65, 0.97)	All-cause: Only post diagnosis RR 0.68 (0.61, 0.92). Pre and post RR 0.95 (0.71, 1.28)
Coghill et al [22]	56/346	Cause specific	37, 72	HR 0.76 (0.61, 0.95)	
Din et al [23]	354/526	Cause specific	125, 761	OR 0.78 (0.65, 0.92)	Aspirin result; also data on NSAIDs ^a^
Domingo et al [24]	125/761	Recurrence	22, 174	HR 0.86 (0.55–1.35)	Wild and mutated combined
		All-cause		HR 0.88 (0.53, 1.47)	
Fuchs et al [25]	72/830	Recurrence or death	N.A.	HR 0.48 (0.24, 0.99)	Compared with non-consistent use. Consistent users: HR 0.45 (0.21, 0.97) for disease recurrence
		All-cause		HR 0.52 (0.19, 1.46)	
Goh et al [26]	92/726	Specific	21, 160	HR 0.71 (0.43, 1.16)	Death or recurrence 0.38 (0.17, 0.84). Benefit only after 5 years)
Liao et al [27]	155/395	Specific	68, 122	HR 0.83(0.61–1.23)	Wild and mutated combined
	403/964	All-cause		HR 0.87 (0.71, 1.06)	
McCowan et al [28]	894/2980	Specific	420, 601	HR 0.58 (0.45, 0.75)	
		All-cause	897, 1101	HR 0.67 (0.57, 0.79)	
Ng et al [29]	75/724	Recurrence or death	19, 214	HR0.68 (0.42, 1.11)	Consistent aspirin HR 0.51 (0.28, 0.95)
		Overall mortality	14, 146	HR 0.63 (0.35, 1.12)	
Reimers et al [30]	178/784	Overall mortality	68, 380	RR 0.67 (0.52, 0.88) ^c^	HLA class 1 antigen groups amalgamated
Sun et al [31]	?/931	Cancer specific survival	931 total events	HR 0.77 (0.52, 1.14)	CTNNBI mutated and non- mutated groups combined
Walker et al [32]	2619/13,994	All-cause	192, 3910	HR 0.91 (0.82, 1.00)	No aspirin pre diagnosis: HR 0.99 (0.84, 1.16); aspirin pre diagnosis: HR 0.86 (0.76, 0.98)
Zanders et al [33]	490/	All-cause	N.A.	HR 0.98 (0.93, 1.03)	Diabetic patients
**Colorectal specific mortality: HR 0.71** (0.58, 0.87) *heterogeneity p = 0*.*0005*					
**With Bains omitted** ^b^**: HR 0.76** (0.66, 0.88) *heterogeneity p = 0*.*035*					
**All-cause mortality: HR 0.80** (0.70, 0.92) *heterogeneity p = 0*.*0005*; *no omission removes significant heterogeneity*					
**Breast cancer**					
Barron et al [34]	764/4540	Specific	50, 311	HR 0.98 (0.74, 1.30)	Selected de-novo aspirin users
		Total	311,495	HR 1.11 (0.83, 1.50)	
Blair et al [35]	254/591	Specific	26, 22	HR 0.53 (0.30,-0.93)	Selected overweight women
		All-cause	57, 44	HR 0.53 (0.36–0.79)	
Bowers et al [36]	159/440	Recurrence	N.A.	OR 0.48 (0.22, 0.98)	NSAIDs, 81% of which are stated to be aspirin ^a^
Cronin-Fenton et al [37]		Recurrence	N.A.	HR 1.0 (0.90, 1.1)	Conference report
Frazer et al [38]	815/1802	Specific	252, 563	HR 0.42 (0.31–0.55)	
		All-cause	577, 1225	HR 0.53 (0.45–0.63)	
Holmes et al. [39]	?4164/11416 person/years	Specific	109, 56	RR 0.36 (0.24–0.54)	
		All-cause		RR 0.54 (0.41–0.70)	
Holmes et al [40]	1661 cases 3322 controls	Specific	395, 750	HR 0.96 (0.80, 1.16)	
Kwan et al [41]	270/2292	Recurrence	41, 209	RR 1.09 (0.74, 1.61)	
Murray et al [42]	262/1435	Specific	262, 1435	OR 1.00 (0.71, 1.41)	‘High’ dose aspirin 0.94 (0.48, 1.84), but dose imprecise
Wernli et al [43]	7 breast cancer deaths	Specific	7, 141	HR 0.64 (0.27, 1.37)	
	37 total deaths	All-cause	37, 383	HR 0.91 (0.65, 1.29)	
**Breast specific mortality: HR 0.69** (0.46, 1.02), *heterogeneity p<0*.*0005*					
**With Frazer omitted** ^b^**: HR 0.87** (0.69, 1.09), *heterogeneity p = 0*.*186*					
**All-cause mortality: HR 0.73** (0.49, 1.08), *heterogeneity p<0*.*0005; no omission removes significant heterogeneity*					
**Prostate cancer**					
Assayag et al [44]	801/1793	Specific	801, 992	1.46 (1.29, 1.65)	Aspirin only after diagnosis: HR 1.84 (1.59, 2.12) cause specific; HR 1.70 (1.53, 1.88) all-cause. Aspirin also before diagnosis: HR 0.97 (0.81. 1.16) cause specific; HR 0.98 (0.87, 1.18) all-cause
	1686/3502	All-cause	1816, 1686	1.37 (1.26, 1.50)	
Caon et al [45]	917/3851	Cause specific	194, 904	HR 0.91 (0.65, 1.28)	
Choe et al [46]	1817/5552	Cause-specific	36, 298	HR 0.43 (0.21–0.87)	
Daugherty et al [47]	136	Cause specific	136 total	HR 0.77 (0.48, 1.25)	Advanced disease: HR 0.37 (0.15, 0.92). Localised disease: HR 0.86 (0.47, 1.58)
Dhillon et al [48]	N.A.	Cause specific	177 total	HR 1.08 (0.76–1.54)	
Flahavan et al [49]	1131/2936	Cause-specific	N.A.	HR 0.88 (0.67, 1.15)	High aspirin: HR 0.73 (0.51, 1.05)
Gryll et al [50]	504/3165	Cause-specific	504, N.A.	HR 0.94 (0.78, 1.14)	
Jacobs Chun et al [51]	41/74	All-cause	6, 8	HR 0.44 (0.15–1.28)	High risk patients selected
Jacobs, Newton et al [52]	301/7118	Cause specific	134, 112	HR 0.98 (0.74, 1.29)	In high risk patients: HR 0.60 (0.37, 0.97)
Stock et al [53]	453/1,619	Cause specific	115, 338	HR 1.03 (0.79, 1.34)	Survival after 5yrs of NSAIDs: HR 0.54 (0.26, 1.13)
**Prostate specific mortality: HR 0.94** (0.76, 1.17), *heterogeneity p<0*.*0005*					
**With Assayag omitted** ^b^**: HR 0.89** (0.79, 0.99) *heterogeneity p = 0*.*261*					
**All-cause mortality**: **HR 0.89** (0.30, 2.61), *heterogeneity p = 0*.*04*, *Only two studies*					
**Other cancers**					
Nagle et al [54]	N.A. (Ovarian)	Overall survival	115,338	HR 0.92 (0.81. 1.06)	Aspirin plus NSAIDs
Fontaine et al [55]	412/1,765 (Lung)	Survival	N.A.	HR 0.84	
Pastore et al [56]	574 (Bladder)	Recurrence	42,98	OR 0.75 (0.45, 1.24)	Effect of aspirin negated by statins
Chae et al [57]	536 (Mix of female cancers)	Survival	N.A.	HR 0.82 (0.57, 1.18)	PIK mutation: HR 0.59 (0.35, 0.98).Wild type: HR 1.80 (1.01, 3.23)
Chae et al [58]	280 (chronic lymphocytic leukaemia)	Survival	N.A.	HR 0.40 (0.21, 0.79).	Aspirin + statins together
MacFarlane et al [59]	416/779 (head & neck)	Survival	178/331 all-cause deaths	HR 0.56 (0.44, 0.71)	Post-diagnostic aspirin
	387/810 (Oesophagus)	Survival	209/756 all-cause deaths	HR 0.54 (0.45. 0.64)	

CI: confidence interval; HR: hazard Ratio; N.A.: not available; NSAID: non-steroidal anti-inflammatory drug; OR: odds ratio; RCT: randomised controlled trial; RR: risk ratio.

^a^The inclusion of NSAIDs other than aspirin posed difficulties but we assumed that aspirin was the major drug used, and evidence for this is given in one of the studies [36].

^b^The examination of heterogeneity by the omission of papers was based on sensitivity analyses. The Newcastle-Ottawa grade of Frazer et al [38] and Assayaq et al [44] were both 9/10 and correspondence with the authors revealed no likely reason the heterogeneity. The data for Bains et al (Grade 7/10) is taken from a poster presentation and details of adjustments for confounding appear to be limited. Attempts to correspond with the author failed.

^c^Reimers [30] reported a risk ratio and could not be included in the meta-analysis.

The sixteen reports of patients with colorectal cancer [8,19–33] give a pooled hazard ratio (HR) of 0.71 (95% CI 0.58, 0.87) for cause specific mortality (11 reports) and there is marked heterogeneity. Sensitivity analyses identified Bains [20] and when omitted there is reduced heterogeneity, and the HR is 0.76 (95% CI 0.66, 0.88). Three of these studies give data for the effect of aspirin in the proximal and the distal colon separately. Two of them [21,22] are homogeneous and combining them gives: HR 0.74 (95% CI 0.52, 1.04) for proximal colon and HR 1.03 (95% CI 0.78, 1.35) for distal colon. The third study [26] shows that compared with the effect in the distal colon, aspirin was associated with an HR of 0.84 (95% CI 0.56, 1.24) for cancer in the proximal colon. Pooled data for all-cause mortality are shown in the Table.

Data for ten breast cancer studies [34–43] are shown in Table 3, and a pooled HR on the effect of aspirin on cause-specific deaths cancer mortality in five studies is 0.69 (95% CI 0.46, 1.02). There is significant heterogeneity (P<0.0005), but on omitting a paper identified in sensitivity analysis [38] the heterogeneity is reduced (P = 0.19) and the HR becomes (0.87; 95% CI 0.69, 1.09). Data on all-cause mortality is given in the Table.

Amongst ten studies of aspirin and prostate cancer [44–53] nine give a cause specific mortality of 0.94 (95% CI 0.76, 1.17) with significant heterogeneity, Sensitivity analyses indicated that one study is responsible [44] and its omission led to an HR of 0.89 (95% CI 0.79, 0.99) and heterogeneity p = 0.26.

Six studies of other cancers [54–59] are included in Table 3. Benefit from aspirin is suggested in all six, but they are too diverse to justify meta-analysis.

**Fig 2:**
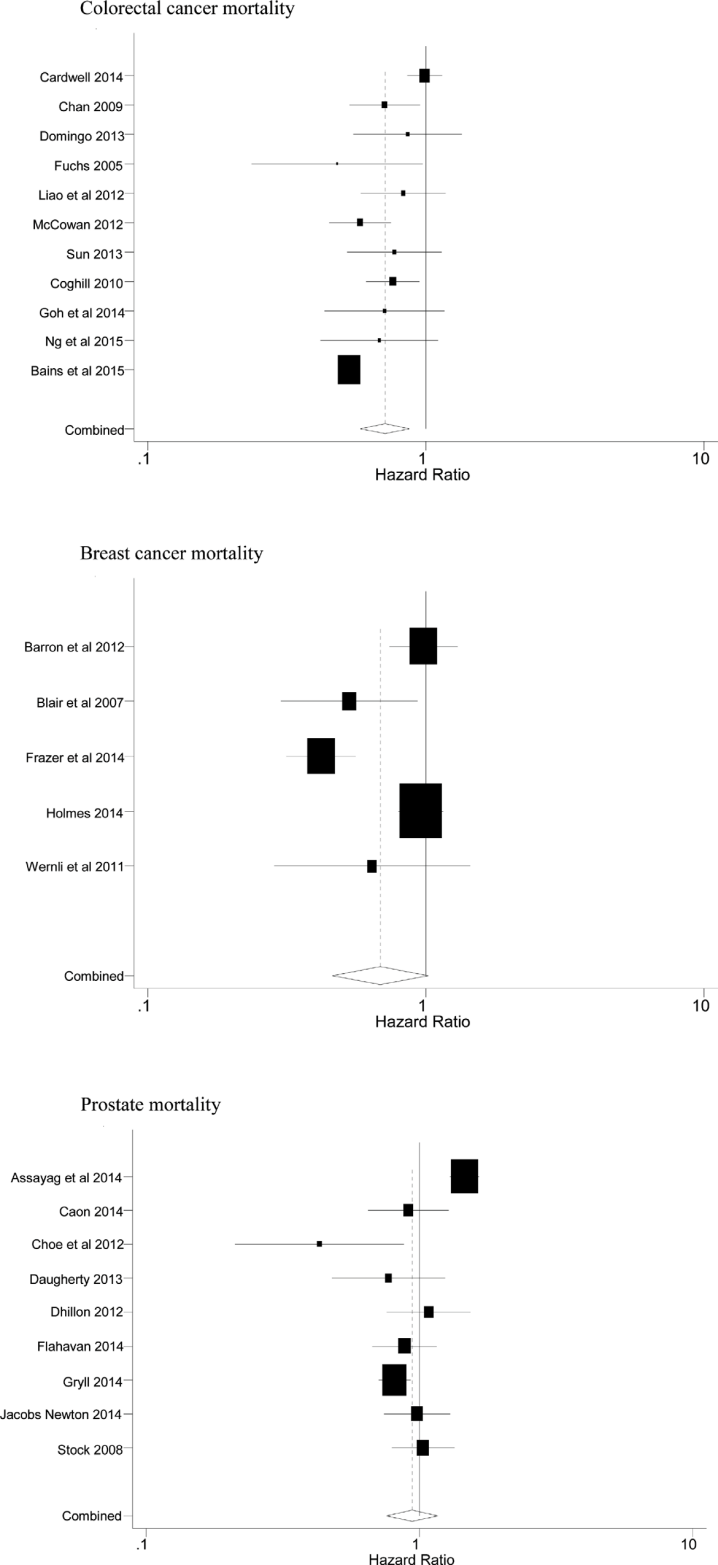
Forest plots of the cause specific mortality summarised in Table 3.

**Fig 3:**
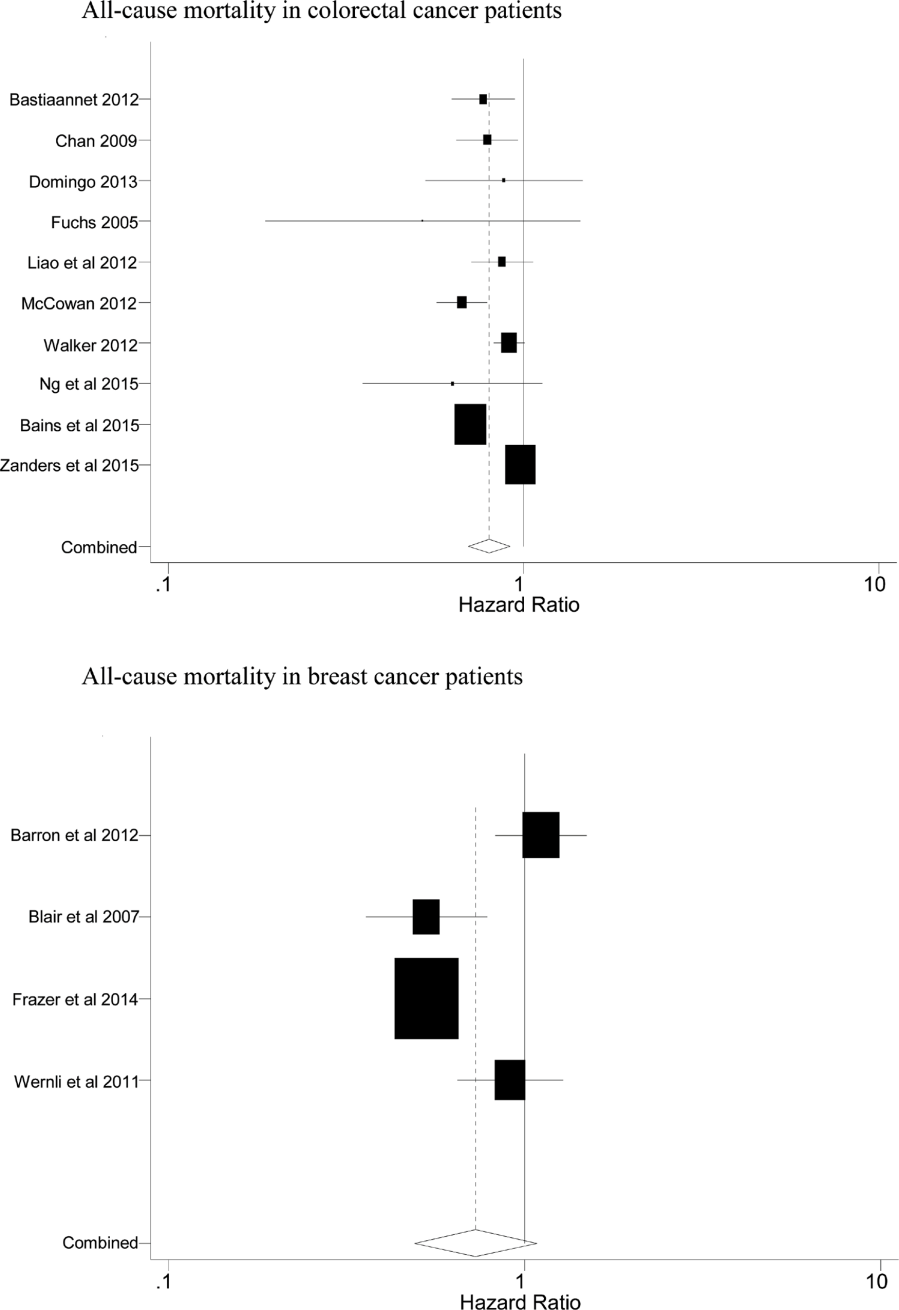
Forest plots of the all-cause mortality summarised in Table 3.

A few of the reports give details of grade or stage of the cancer, but these were too few to enable any relevant analyses in relation to the effect of aspirin. Later however we quote a few comments on aspirin and ‘advanced’ cancer. Figs 2 and 3 respectively show the Forest plots of the cause specific mortality and the all-cause mortality (although only two reports [44,52] stated all-cause mortality in patients with prostate cancer).

It is possible to examine the pooling of the HRs for the three main cancers, and it seems not unreasonable to do this because the various pairs of HRs do not differ significantly (thus: for colon and breast cancer P = 0.90; for colon and prostate cancer P = 0.06 and for breast and prostate cancers P = 0.18). An overall meta-analysis for cause-specific mortality from these three cancers is 0.78 (95% CI 0.66, 0.92). Naturally, this has to be accepted with great caution, particularly as there is significant heterogeneity (P<0.0005), even though the omission of the three papers already identified as ‘outliers’ by sensitivity analysis [20,38,40] reduces the heterogeneity (P = 0.03) and gives an overall HR of 0.83 (95% CI 0.76, 0.90). Egger’s test for publication bias [13] is not significant (P = 0.30).

### Aspirin and metastatic spread

An effect of aspirin on metastatic spread is clearly an evidence of treatment and the data in Table 4, although sparse, are therefore of considerable importance. Two studies of breast cancer [14,46], two of prostate [23,38] and one of both cancers together with colon [4], give evidence of a reduction in spread by aspirin. A combined estimate gives a relative risk for aspirin of 0.77 (95% CI 0.65, 0.92), though there is significant heterogeneity between the studies.

**Table 4:** Aspirin and metastatic spread in observational studies.

Study	Cohort	Numbers (aspirin, no aspirin)	Cancer	Reduction (95% CI)	Comment
Algra & Rothwell [4]	150 Case-control and 45 cohort studies	141577 in case-control 41575 in cohorts	All cancers	RR 0.71 (0.60, 0.84)	No reduction in localised spread: OR 0.98 (0.88–1.09)
Choe et al [46]	Selected patients from a cancer centre	2175, 3780	Prostate	RR 0.50 (0.37–0.68)	
Jacobs, Chun et al [51]	Series of patients	45, 29	Prostate	RR 0.42 (0.12, 1.45)	Reported as 12.2% vs. 26.7%, P = 0.039 at 5 years
Barron et al [62]	Ireland National Cancer Register	740, 2056	Breast	RR 0.89 (0.81. 0.97)	Spread to lymph nodes: RR 0.81 (0.68, 0.96) in quarter women with highest aspirin dose
Ljung et al [60]	Nationwide Swedish cohort	N.A.	Breast	RR 0.94 (0.87–1.03)	Anticoagulants; 96% were aspirin
				RR 0.80, (0.50–1.29)	In younger women
				HR 0.84 (0.64, 1.11)	Reduced spread to lymph nodes
**RR 0.77** (0.65–0.92), *heterogeneity p<0.0005, Algra 2012 [4] omitted*					

CI: confidence interval; HR: hazard Ratio; N.A.: not available; OR: odds ratio; RR: risk ratio.

### Aspirin and mutations

A mutation in PIK3CA, a gene which produces a protein that increases Cox-2 and prostaglandin activity, has been shown to enhance the response of the tumour to aspirin. The prevalence of this mutation is stated in several of the present studies as around 15–20% [24,27,61]. Table 5 summarises the relevant data and confirms a marked reduction in mortality in tumours with the mutation (HR 0.45; 95% CI 0.28, 0.71), while it is uncertain if there is benefit from aspirin in patients without the mutation (HR 0.94; 95% CI 0.67, 1.32). This last statement is based on comparison between pairs of HRs using the normal approximation of the difference between log HRs.

**Table 5:** Effect of aspirin: relevance of PIK3CA mutation.

Authors	Cancer	Wild (95% CI)	Mutation/overexposure (95% CI)
**Cause specific mortality**			
Chan et al [8]	Colorectal	HR 1.22 (0.36, 4.18)	HR 0.39 (0.20, 0.76)
Domingo et al [24]	Colorectal	HR 0.94 (0.59, 1.49)	HR 0.11 (0.01, 0.83)
Liao et al [27]	Colorectal	HR 0.90 (0.53, 1.54)	HR 0.28 (0.04, 2.10)
Kothari et al [61]	Colorectal	No patients	HR 0.66 (0.31, 1,38)
**‘wild’ cancers: HR 0.94** (0.67–1.32), *heterogeneity P = 0*.*91*			
**‘mutant’ cancers, HR 0.45** (0.28–0.71), *heterogeneity P = 0*.*40*			
**All-cause mortality**			
Chan et al [8]	Colorectal	HR 1.05 (0.55, 2.02)	HR 0.62 (0.42, 0.93)
Domingo et al [24]	Colorectal	HR 0.95 (0.56, 1.61)	HR 0.29 (0.04, 2.33)
Liao et al [27]	Colorectal	HR 0.97 (0.68, 1.37)	HR 0.59 (0.24, 1.41)
Kothari et al [61]	Colorectal	No patients	HR 0.95 (0.55, 1.63)
Chae et al [57]	Several cancers	HR 1.80 (1.01, 3.23)	HR 0.75 (0.17, 3.20)
**‘wild’ cancers: HR 1.10** (0.84–1.44), *heterogeneity P = 0*.*31*			
**‘mutant’ cancers, HR 0.69** (0.52–0.93), *heterogeneity P = 0*.*66*			

CI: confidence interval; HR: hazard ratio.

Evidence on these or other mutations appears not to be available in the present studies of breast or prostate cancers.

### Aspirin taken before a cancer diagnosis

Does aspirin treatment affect cancers which have developed while aspirin has been taken? It may be that cancers which develop while aspirin is being taken are less responsive to the effect of aspirin. Table 6 summarises relevant data and shows that aspirin taken before the diagnosis of cancer is of little or no relevance to the treatment effect.

**Table 6:** Aspirin also taken prior to diagnosis.

Author	Cancer	Aspirin only after diagnosis, not before (95% CI)	Aspirin after and before diagnosis (95% CI)
**Cause specific mortality**			
Chan et al [8]	Colorectal	HR 0.53 (0.33, 0.86)	HR 0.89 (0.59, 1.35)
Coghill et al [22]	Colorectal	HR 0.77 (0.58, 1.00)	HR 0.75 (0.56, 1.00)
Goh et al [36]	Colorectal	HR 0.81 (0.51, 1.28)	HR 1.06 (0.71, 1.58)
Cardwell et al [21]	Colorectal	HR 1.08 (0.71, 1.63)	HR 0.75 (0.43, 1.29)
Liao et al [27]	Colorectal	HR 0.83 (0.50–1.39)	HR 0.79 (0.49–1.27)
Barron et al [62]	Breast	HR 0.99 (0.68, 1.46)	HR 0.80 (0.62, 1.04)
Kwan et al [41]	Breast	RR 1.23 (0.72, 2.11)	RR 0.99 (0.60, 1.64)
Assayag et al [44]	Prostate	HR 1.84 (1.59, 2.12)	HR 0.97 (0.81. 1.16)
Jacobs/Newton et al [52]	Prostate	HR 0.97 (0.65, 1.45)	HR 1.04 (0.73, 1.47)
**Aspirin before & after diagnosis: HR 0.84** (0.70–1.00). *Heterogeneity*: *p = 0*.*70 (colorectal cancer studies only)*			
**Aspirin only after diagnosis: HR 0.79** (0.65–0.97). *Heterogeneity*: *p = 0*.*30 (colorectal cancer studies only)*			
**All-cause mortality**			
Bastiaannet et al [19]	Colorectal	HR 0.70 (0.57, 0.88)	HR 0.88 (0.83, 0.94)
Chan et al [8]	Colorectal	HR 0.68 (0.51, 0.92)	HR 0.96 (0.71, 1.28)
Walker et al [32]	Colorectal	HR 0.99 (0.84, 1.16)	HR 0.86 (0.76, 0.98)
Goh et al [26]	Colorectal	HR 0.86 (0.58, 1.27)	HR 1.04 (0.72, 1.48)
Liao et al [27]	Colorectal	HR 0.91 (0.66–1.26)	HR 0.81 (0.58–1.12)
Barron et al [62]	Breast	HR 1.11 (0.83, 1.50)	HR 0.81 (0.66, 0.99)
Assayag et al [44]	Prostate	HR 1.69 (1.53, 1.88)	HR 0.99 (0.87, 1.18)
Macfarlane et al [59]	Oesophagus	HR 0.84 (0.97, 1.26)	HR 1.11(0.97, 1.26)
**Aspirin before and after diagnosis: HR 0.88** (0.83–0.93). *Heterogeneity*: *p = 0*.*82 (colorectal cancer studies only)*			
**Aspirin only after diagnosis: HR 0.83** (0.69–0.98) *Heterogeneity*: *p = 0*.*06 (colorectal cancer studies only)*			

CI: confidence interval; HR: hazard ratio.

### Advanced disease and high risk groups

Data in three reports of prostate cancer suggest that the effect of aspirin may be greater in advanced disease. Thus Daugherty et al [47] describe an effect of aspirin in ‘advanced’ prostate cancer (HR 0.37; 95% CI 0.15, 0.92) which is greater, than in localized disease (HR 0.86; 95% CI 0.47, 1.58) Similarly Jacobs, Newton et al [52] report an HR of 0.60 (95% CI 0.37, 0.97) in ‘high-risk’ patients, contrasted with the effect of aspirin in the total cohort (HR 0.98; 95% CI 0.74, 1.29) in the total series of patients. Neither of these differences are however significant, nor is a result reported by Jacobs, Chun et al [51] who selected ‘high risk’ patients and reported a reduction by aspirin HR 0.44; (95% CI 0.15, 1.28).

### The dose and consistency of aspirin taking

Many of the reports state, or imply that aspirin at a dose appropriate for vascular protection had been used and only a very few reports comment further. Several studies report greater effects with ‘high’ dose aspirin [49,63] though no difference is significant.

Several authors refer to the consistency of aspirin taking. Chan et al [8] give evidence consistent with a gradient (P < 0.04), the maximum benefit being with more than five aspirin tablets per week. Baastinnet et al [19] reported that the benefit of aspirin in all who took the drug as HR 0.77 (95% CI 0.63, 0.95), whereas ‘frequent’ use was associated with a possible slight increase in protection (HR 0.70; 95% CI 0.57. 0.88). Ng et al [29] reported HR 0.51 (95% CI 0.28, 0.95) for consistent use, compared with HR 0.68 (95% CI 0.42, 1.11) in the total cohort. Fuchs et al [25] state that compared with non-consistent use, ‘consistent’ users had a much greater reduction (HR 0.45; 95% CI 0.21, 0.97).

Several authors state that an effect of aspirin because apparent only after 3–5 years of therapy. Goh et al [26] state that they found evidence of benefit only after 5 years, Stock et al [53] who reported no benefit to prostate cancer overall (HR 1.03; 95% CI 0.79, 1.34) states that after five years of aspirin taking there was benefit (HR 0.54; 95% CI 0.26, 1.13) and an effect of aspirin taking in the study of lung cancer [55] became significant only after three years (HR = 0.84; CIs not stated).

### Aspirin and bleeding

An excess of bleeding attributable to aspirin has been well studied in short-term vascular trials [64,65]. It is however appropriate to ask whether or not the risk of bleeding attributable to aspirin is similar in patients with cancer to that reported from the vascular trials. A few reports in the present series give a measure of reassurance on this. Din et al [23] who examined NSAID use state that there were no major bleeding complications. Liu et al [18] state that no side effects caused by aspirin were noted in any patient in the study. Curigliano et al [66] examined short-term aspirin taking by patients with breast cancer and stated that no major bleeding complication occurred.

The corresponding authors of the other reports in this series were written to. Replies received from twenty-one authors stated that no data on bleeding had been recorded.

## Discussion

The evidence we present from a systematic overview of the literature gives support to the use of aspirin as an additional treatment of cancer. The evidence is limited, and while it is encouraging in the case of bowel cancer, there is insufficient evidence to dismiss a role for aspirin as an adjunct treatment of cancers other than colorectal. In fact, its use can be justified on the basis of its likely benefit on outcomes other than death, including its probable reduction in metastatic spread and its reduction in vascular disease events, including venous thromboembolism.

Differences between individual studies leading to significant heterogeneity is to be expected in any collection of observational studies such as those we present, and it does limit confidence in the results. However, if, for each of the three cancers, an out-lying study identified by detailed sensitivity analyses is omitted, heterogeneity is reduced to an acceptable level and for each cancer there is evidence suggestive of reductions in mortality and in metastatic spread.

In colon cancer there is evidence of a reduction in colorectal deaths of about 25%, and perhaps about 20% in All-cause mortality. If one report [13] is omitted the evidence of benefit in breast cancer is of a possible 13% reduction in cause-specific deaths, and for prostate cancer a possible reduction of perhaps about 11%.

With the present level of evidence, the pooling of data for the three cancers would seem to be not unreasonable and following omissions of three outliers, unacceptable heterogeneity is resolved. A meta-analysis then suggests a possible overall reduction by aspirin of about 15% (pooled HR 0.83; 95% CI 0.76–0.90). The evidence of a reduction in metastatic spread (RR 0.77; 95% CI 0.65–0.92) gives further encouragement to the use of therapeutic aspirin in cancer while awaiting evidence from *ad hoc* randomised trials.

It would be unreasonable to attempt to draw firm conclusions from the single studies on lung cancer [31], on oesophageal cancer [45], and on lymphatic leukaemia [22]. However the suggestive benefit in these studies, together with that reported for a mix of colon and women’s cancer [21] indicates an urgent need for more observational studies in the less common cancers, some of which may never be subjected to evaluation in randomised trials. It would also be helpful if in the reporting of new studies information on the stage and grade etc. of the cancers could be indicated.

The evidence we present on the biomarker PIK3CA has been confirmed in an overviews by other authors [67], and similar data on other markers have been shown by other authors [68]. Thus Sun et al [31] who examined CTNNB1, a gene associated with cell adhesion and of relevant to familial polyposis, reported a marked enhancement in the effect of aspirin (HR 0,53; 95% CI 0.30, 0.95) compared with the effect in patients with the wild gene (HR 1.06; 95% CI 0.62, 1.83). A different approach to this issue was adopted by Chan et al [8] who used an overexpression of COX-2 in the primary tumour as an indication of a relevant mutation, and showed that overexpression was associated with reductions in colon cancer mortality (HR 0.39; 95% CI 0.20, 0.76) compared with the effect in other patients (HR 1.22; 95% CI 0.36, 4.18).

All this suggests that the reduction by aspirin may be restricted to patients whose tumours show mutation in PIK3CA, HLA class I antigen, or show COX-2 over-expression. In colorectal cancer these subgroups represent approximately 17%, 54% and 50% of all patients, and our data suggest a reduction of about 50% in colorectal mortality, though another overview [67] suggested a reduction of only about 30%, while neither overview showed any reduction in those without the mutation. The scarcity of evidence on mutation in cancers other than colon is most unfortunate [69].

And yet any selection of patients for treatment with aspirin on the basis of a mutation or any other marker of cancer risk would be totally unwarranted on present evidence. Metastases are a major source of pain and other undesirable effects in solid cancers [70,71] and perhaps 90% of cancer deaths are at least in part due to metastases [72] and the withholding of aspirin would deny these possible benefits. Furthermore, the risk factors for vascular disease overlap with those for cancer and the withholding of aspirin would also deny patients the vascular benefits of aspirin, including the possible reduction of the excess risk of venous thromboembolism during chemotherapy. In fact, the marked increase in the risk of venous thrombosis in patients with cancer [66,73], has been shown to be reduced by low-dose aspirin [74], and it has therefore been recommended that prophylactic anticoagulants should be considered in all patients with cancer [75].

A major uncertainty in what we report arises from the possible omission of relevant reports, together with publication bias, and the test we performed suggests that this last may have occurred (Egger's test [13] P = 0.037). Furthermore, underlying all observational studies is the issue of residual confounding, and while this cannot be dismissed, it seems unlikely to have operated to any important degree as all the studies reviewed included multivariate adjustments.

On the other hand certain time biases could be present in some of the studies, and especially in retrospective case-control studies [76]. Patients not taking aspirin at the time of diagnosis can be defined, used as ‘controls’ and followed thereafter. Patients who start taking aspirin after receiving a diagnosis of cancer cannot be identified as ‘cases’ until they start taking the drug. It is possible that these will be identified later than the ‘control’ patients, and they will therefore be observed and deaths identified during a shorter time that that during which the patients not taking aspirin are observed. This has been called an ‘immortal’ time bias and Assayag & Azolay [44] include a detailed discussion of it.

All the reports in the present series were examined and while immortal time bias cannot be dismissed with certainty, an important effect upon the overall estimates of the effect of aspirin seems most unlikely. In fact, there is little difference in the overall mortality of the patients who had taken aspirin before diagnosis, and (presumably) continued to take it after diagnosis (HR 0.92), and the patients who had not taken aspirin before diagnosis (HR 0.90), in whom there could have been a time lag and thus, an ‘immortal time bias’ (see Table 6).

While a serious limitation in the present evidence is that little comes from randomised trials, yet evidence from further observational studies is urgently needed to evaluate more fully how patients likely to benefit from aspirin can be identified. In particular evidence on PIK3CA, other mutations and other possible markers should be collected as a matter of urgency in cancers of breast, prostate and other organs. The results of such studies should be made available for the encouragement and guidance of colleagues setting up randomised trials, and, in fact, the further question arises whether or not these mutations are of relevance to aspirin used in prophylaxis.

The possible benefits of aspirin must of course be evaluated against it side effects. Shortly after aspirin taking commences the risk of a gastrointestinal (GI) bleed is high but the risk falls rapidly thereafter [77,78], and in short-term trials the additional risk of a bleed from low-dose aspirin amounts to perhaps one or perhaps two patients in every 1,000 on low-dose aspirin [64,65]. After about three years of aspirin taking however, there appears to be no evidence of any excess GI bleeds attributable to the drug [78]. Moreover, the incidence of GI bleeding is highly sensitive to the presence of gastric pathology [78,79], and careful enquiries should therefore be made about current or past gastric symptoms, and about a high alcohol consumption [80]. The use of a gastroprotective drug together with the aspirin should be carefully considered if pathology is suspected.

The most serious bleeds are those that lead to death, and despite frequent references to fatal bleeds attributed to aspirin, there appears to be no valid evidence that deaths from GI bleeds are increased by low-dose aspirin [81]. In a recent study of patients admitted to hospital with ‘gross’ GI bleeding [82] the hospital stay of patients who had been taking aspirin was significantly shorter than that of patients who had not been on aspirin, and no patient whose bleed had been attributed to aspirin experienced an uncontrolled haemorrhage or died due to excessive bleeding.

Cerebral bleeds attributable to aspirin are rare, about one or two per 10,000 patient-years. Hypertension is the major factor in such bleeds [2] and in a randomised trial of aspirin based upon patients with hypertensive disease all of whom were adequately treated with anti-hypertensive drugs, there was the same number of cerebral bleeds in ten thousand patients on aspirin (19 patients) as in ten thousand on placebo (20 patients) [83]. A reduction in the risk of a cerebral bleed is therefore likely if the blood pressure of every person starting aspirin is checked, and adequately treated if raised [84].

### Other overviews

An early overview of studies of aspirin and cancer was based on three small randomised trials and two observational trials, and this led to the conclusion: ‘aspirin may have a role in the adjuvant setting… and should not be overlooked [9]. A further overview of three studies of colorectal, one of breast, two of prostate and one of oesophageal cancers judged that aspirin decreases the development and spread of metastases [10].

Li et al [85] reported a systematic review and meta-analysis of seven studies of colorectal cancer. Aspirin use after a diagnosis of colon cancer was reported to reduce overall all-cause mortality (HR 0.84; 95% CI 0.75, 0.94), but if aspirin had been taken before cancer benefit was uncertain (HR 0.77; 95% CI 0.52, 1.14). Ye et al [86] conducted a meta-analysis of seven of the studies of colon cancer in the present review, and reported effects of aspirin on both colon cancer mortality and overall mortality very close to what we report.

Huang et al [87] identified 16 studies in which a NSAID or aspirin had been used in patients with breast cancer. On the basis of meta-analyses they reported that aspirin taken after diagnosis, but not before, was associated with improved breast cancer survival (HR 0.69; 95% CI 0.50, 0.96). Zong et al [88] also reported a systematic review and meta-analysis of breast cancer patients in eight cohort studies and two nested case-control studies, and judged that post-diagnostic aspirin was associated with a significant reduction in the relative risk of death from breast cancer (RR 0.84; 95% CI 0.63, 1.12). Liu et al [89] conducted a systematic review and identified 39 studies of NSAIDs, including aspirin. A meta-analysis of prostate specific mortality in seven studies of aspirin gave an HR = 0.86 (95% CI 0.78, 0.96) and the effect of aspirin in these studies were judged to be more consistent than those for other NSAIDs.

## Conclusions

It appears likely that low-dose aspirin has a beneficial role as an adjunct treatment of cancer. Reductions in mortality are shown in colon cancer, probably in prostate cancer and possibly in breast and individual studies of several other cancers also suggest benefit. Aspirin benefit in colorectal cancers, and possible other cancers, may be restricted to patients with tumours expressing certain genetic mutations. However, other benefits of low-dose aspirin, including reductions in metastatic spread and in vascular events, including venous thromboembolism appear to be independent of these biomarkers, and so information on aspirin should be given to patients whatever the state of the possible biomarkers.

The heterogeneity within the currently available studies–both between different cancers, and within the different studies of each cancer, together with evidence suggesting some publication bias, are such that further evidence from a number of adequately powered randomised, placebo controlled trials is urgently required, including trials of less common cancers. Evidence on the possible role of aspirin in uncommon cancers, and the possible enhancement of its effect if mutation and other markers of increased sensitivity to the actions of aspirin, are also urgently needed. Much of this evidence could some from further observational studies.

Nevertheless, despite the need for randomised trials, we believe the evidence of benefit from aspirin is sufficiently persuasive that physicians should engage with patients in a presentation and discussion of aspirin as an additional treatment. Furthermore, we hold that patients should be given this evidence within the context of a healthy lifestyle [90], they should be allowed to make their own decision about aspirin therapy, and should then be supported in whatever decision they make [91].

## Supporting Information

S1 Table PRISMA checklist. (DOC)
